# Is conservation based on best available science creating an ecological trap for an imperiled lagomorph?

**DOI:** 10.1002/ece3.7104

**Published:** 2020-12-19

**Authors:** Amanda E. Cheeseman, Jonathan B. Cohen, Sadie J. Ryan, Christopher M. Whipps

**Affiliations:** ^1^ Department of Environmental and Forest Biology SUNY College of Environmental Science and Forestry Syracuse NY USA; ^2^ Quantitative Disease Ecology and Conservation (QDEC) Lab Department of Geography University of Florida Gainesville FL USA; ^3^ Emerging Pathogens Institute University of Florida Gainesville FL USA; ^4^ School of Life Sciences University of KwaZulu‐Natal Durban South Africa

**Keywords:** adaptive management, density, eastern cottontail, New England cottontail, shrubland, survival

## Abstract

Habitat quality regulates fitness and population density, making it a key driver of population size. Hence, increasing habitat quality is often a primary goal of species conservation. Yet, assessments of fitness and density are difficult and costly to obtain. Therefore, species conservation often uses “best available science,” extending inferences across taxa, space, or time, and inferring habitat quality from studies of habitat selection. However, there are scenarios where habitat selection is not reflective of habitat quality, and this can lead to maladaptive management strategies. The New England cottontail (*Sylvilagus transitionalis*) is an imperiled shrubland obligate lagomorph whose successful recovery hinges on creation of suitable habitat. Recovery of this species is also negatively impacted by the non‐native eastern cottontail (*Sylvilagus floridanus*), which can competitively exclude New England cottontails from preferred habitat. Herein, we evaluate habitat quality for adult and juvenile New England and eastern cottontails using survival and density as indicators. Our findings did not support selection following an ideal free distribution by New England cottontails. Instead, selected resources, which are a target of habitat management, were associated with low survival and density and pointed to a complex trade‐off between density, survival, habitat, and the presence of eastern cottontails. Further, movement distance was inversely correlated with survival in both species, suggesting that habitat fragmentation limits the ability of cottontails to freely distribute based on habitat quality. While habitat did not directly regulate survival of juvenile cottontails, tick burden had a strong negative impact on juvenile cottontails in poor body condition. Given the complex interactions among New England cottontails, eastern cottontails, and habitat, directly assessing and accounting for factors that limit New England cottontail habitat quality in management plans is vital to their recovery. Our study demonstrates an example of management for possible ecological trap conditions via the application of incomplete knowledge.

## Graphical abstract

Habitat selection studies are frequently used to infer habitat quality and establish management guidelines; however, habitat selection may become decoupled from fitness, resulting in incorrect inferences of habitat quality. We evaluate habitat quality using survival and density as indicators for New England cottontails and non‐native eastern cottontails. Our findings suggest New England cottontails selected resources that were associated with low survival and density, and pointed to a complex trade‐off between density, survival, habitat, and the presence of eastern cottontails.

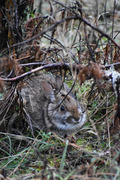

## 1 INTRODUCTION

It is broadly recognized that the availability and quality of habitat influences wildlife density, survival, and reproduction and, as a result, strongly regulates population size (Heinrichs et al., 2016; Johnson, 2007). In fact, habitat loss and degradation are frequently cited as the leading causes of species declines globally (Butchart et al., 2006; Gibbons et al., 2000; Tilman et al., 2017). Thus, management to create habitat and improve habitat quality is often a primary focus of conservation efforts. These efforts have had notable success in recovering declining wildlife populations by improving survival and reproductive rates and increasing population size (Catlin et al., 2011; Innes et al., 1999; Warren, 1991; Whitehead et al., 2008). However, there are also many instances wherein management efforts were ineffective or even detrimental to target species, so much so that ecological restoration is considered among the top three anthropogenic causes of ecological traps (Armstrong et al., 2007; Ausden et al., 2001; Robertson et al., 2013). These failures are generally attributed to overlooking key factors that alter habitat quality, leading to ineffective conservation measures and a waste of limited conservation funding.

In order to enact effective habitat management, a robust understanding of how survival, reproduction, and density vary among patches of differing quality is recommended (Johnson, 2007). However, measures of survival, reproduction, or density are often prohibitively difficult and costly to obtain and management decisions must proceed with the “best available science” (Doremus, 2004). This is particularly true for rare or threatened species given legal mandates to use “best available science” under the U.S. Endangered Species Act and considering their scarcity and low population densities often preclude the effective application of traditional research methods (Murphy & Weiland, 2016). Conservation and management strategies using “best available science” often apply existing information from similar systems or rely on ecological theory to make broader inferences from correlative studies—the latter is commonly the case with habitat selection studies.

Selection for high‐quality habitat evolves due to its effects on individual fitness (Fretwell, 1969), and strong selection for habitat features is evident across diverse taxa (Binckley, 2017; Crosby et al., 2019; Gorosito et al., 2018; Sawyer & Brashares, 2013; White et al., 2019). When species are at equilibrium and can select habitat according to an ideal free distribution, selection is indicative of habitat quality (Jones, 2001; Pulliam & Danielson, 1991). Thus, practitioners often use patterns in species occupancy or resource selection to infer habitat quality and produce management recommendations (Johnson, 2007). Although this approach can be effective, habitat selection may not always be a reliable indicator of fitness and density (Van Horne, 1983). The relationship between survival, reproduction, and density and habitat selection can become decoupled in altered or disturbed systems, resulting in weak relationships between selected resources and fitness or in ecological traps where fitness is negatively impacted by preferentially selected resources (Battin, 2004; Robertson & Hutto, 2006). Inferences obtained from decoupled systems can lead to prioritizing conservation for poor‐quality habitat, which is at best inefficient or at worst can exacerbate declines of imperiled target species (Hale & Swearer, 2017).

Moreover, preferential selection for poor‐quality habitat is not uncommon in wildlife populations. For example, the incorrect use of cues to assess habitat quality can lead to selection for resources that are attractive but detrimental to fitness (Nawrocki et al., 2019; Titeux et al., 2019). Numerous processes can result in decoupled systems, for example, historic competitive interactions (Morris et al., 2000), despotic selection where dominant individuals exclude subordinate individuals to high‐density low‐quality habitat (Fretwell, 1969), or when populations are regulated through density‐dependent mechanisms such as predation (Morris, 2005). Conflicting patterns of apparent habitat selection and fitness can also result where systems are not able to reach equilibrium (Shochat et al., 2002), such as when habitat fragmentation limits the movement capacity of individuals and thus their ability to distribute among patches on the basis of habitat quality (Stamps et al., 2005). These phenomena can result from the introduction of novel resources (Remeš, 2003), competitors (Cole et al., 2005), or predators (Igual et al., 2007) and occur more frequently in human‐disturbed systems, such as in anthropogenically managed shrublands and grasslands (Bock & Jones, 2004; Robertson et al., 2013; Shochat et al., 2005).

**FIGURE 1:**
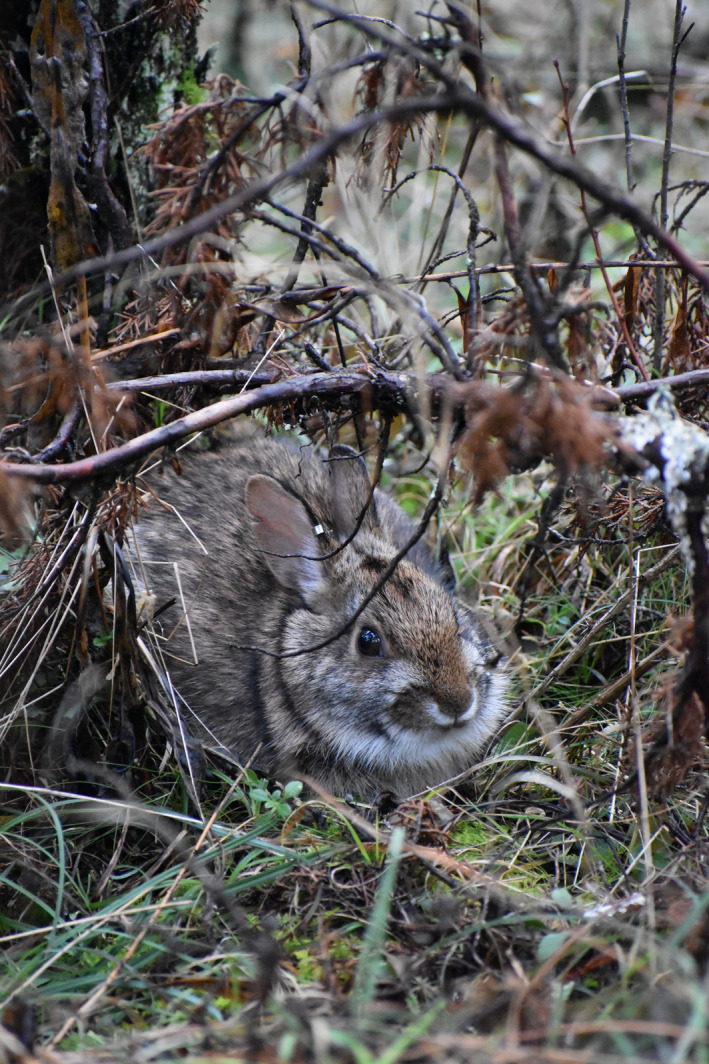
New England cottontail (*Sylvilagus transitionalis*) resting in native‐dominated late successional shrubland

Given the limited availability of conservation funding, it is critical that enacted management is effective at meeting targets. Hale and Swearer (2017) recently highlighted how management can create ecological traps for target species when these actions do not link habitat selection to habitat quality, but they note this possibility has largely been ignored in wildlife research. One species of intense management focus is the New England cottontail (*Sylvilagus transitionalis*, Figure 1), a shrubland obligate lagomorph that has experienced dramatic population declines since the mid‐1900s (Litvaitis et al., 2006). It is listed as vulnerable by the IUCN and was considered for federal listing under the U.S. Endangered Species Act, but denied status as ongoing efforts were deemed sufficient for its recovery (Litvaitis & Lanier, 2019; USFWS, 2015). Declines of New England cottontails are primarily attributed to habitat loss and fragmentation as a result of anthropogenic development and natural forest succession, scramble competition with introduced eastern cottontails over unoccupied areas (*Sylvilagus floridanus*), and the modification of habitat by invasive shrub species (Cheeseman et al., 2018; Litvaitis et al., 2008). These declines have gained regional conservation attention, and recovery plans hinge on the successful creation of 20,000 hectares of habitat (Fuller & Tur, 2012). However, despite considerable conservation effort, the area of occupancy for New England cottontails has declined by an additional 50% in the last decade (Rittenhouse & Kovach, in press), raising concerns about the efficacy of existing management within these highly impacted ecosystems.

Past research addressing habitat quality for New England cottontails has largely focused on studies of habitat use and selection (Brubaker et al., 2014; Buffum et al., 2015; Cheeseman et al., 2018, 2019; Kilpatrick et al., 2013; Litvaitis et al., 2003, 2006; Shea et al., 2019; Tash & Litvaitis, 2007). Demographic studies related to habitat quality have been mostly limited to assessments of survival at their northern range boundary (Barbour & Litvaitis, 1993; Brown & Litvaitis, 1995) where eastern cottontails are still scarce, emphasizing the importance of vegetative cover with high stem densities and the influence of large habitat patches on survival (Barbour & Litvaitis, 1993; Brown & Litvaitis, 1995). As a result, conservation management planning for this species has focused on creating early successional shrublands with a high density of native shrubs and low overstory canopy closure to facilitate a denser understory (New England Cottontail Regional Technical Committee, 2017). Unfortunately, management plans do not currently consider the multifaceted relationships among habitat quality, habitat selection, survival, and density nor how the present landscape context, including possible competitive exclusion by an introduced competitor, habitat modification by invasive shrubs, or dispersal costs in highly fragmented landscapes, might affect these relationships. More recent resource selection studies have supported recommendations to manage for early successional shrubland only where eastern cottontails are not prevalent, demonstrating that New England cottontails select for features typically associated with early successional shrublands such as low canopy closure (Cheeseman et al., 2018). However, these authors also found that where eastern cottontails are prevalent, they displace New England cottontails from early successional shrublands. In areas where eastern cottontails are prevalent, New England cottontails instead select for areas of high canopy closure and moderate to high densities of the invasive shrub Japanese barberry (*Berberis thunbergii*), which are themselves indicative of mid‐ to late successional shrublands (Cheeseman et al., 2018). These findings raise concerns over the quality of later successional shrublands for New England cottontails and the suitability of early successional shrublands where eastern cottontails are sympatric. In this study, we examine how resources and landscape features, cottontail health, and competitor prevalence influence habitat quality for New England and introduced eastern cottontails. We examine the survival and density of each species as indicators of habitat quality and assess the impact of eastern cottontail abundance on New England cottontail density across patches with varying resources.

## 2 METHODS

### 2.1 Study area

We conducted our study at 15 sites in the Hudson Valley region of New York, USA, from December 2013 through April 2017. The climate is temperate, average annual rainfall for this period was 51.8 cm (*SD* = 36.5 cm), and winter snow depth was highly variable within and between years, varying from 0 to 63.5 cm (www.ncdc.noaa.gov). Sites were characterized by different‐aged successional shrublands, persistent forested ericaceous shrublands, and/or persistent forested shrub wetlands. Successional shrubland was classified as early, mid, late, and persistent based on the density and height of shrubs, degree of overstory canopy closure, and dominant shrub species in the case of persistent shrublands following Cheeseman et al. (2018). Successional shrublands were often dominated by invasive multiflora rose (*Rosa multiflora*), Japanese barberry, and Oriental bittersweet (*Celastrus orbiculatus*); and ericaceous shrublands contained *Vaccinium* spp. and mountain laurel (*Kalmia latifolia*) understory and an oak (*Quercus* spp.), hemlock *(Tsuga canadensis*), and/or white pine (*Picea glauca*) overstory. Sweet pepperbush (*Clethra alnifolia*) and swamp azalea (*Rhododendron viscosum*) were common in forested shrub wetlands. Overstory tree species composition within forested wetlands was variable and representative of the general tree communities at the site.

### 2.2 Field methods

From December 2013 to October 2016, New England and eastern cottontails were live‐trapped using box traps baited with apple. Traps were set along adaptive transects spanning site boundaries at approximately 25‐m intervals. Captured cottontails were weighed, and morphological measurements were obtained to determine species identity and age. Each individual was examined for parasites, and the bone protrusion, rump, and loin muscles were examined to assess body condition and scored following Bonanno et al. (2008). For analyses, we collapsed categories into “good” and “poor” body condition where individuals scoring in the lowest category in at least two bodily locations were considered to have “poor” body condition, and all others were considered in “good” body condition. For newly captured individuals, a tissue biopsy was obtained from the ear to confirm species identity through genetic analyses and a blood sample was collected in microhematocrit tubes for a hematocrit assessment. Tissue samples were stored in 100% ethanol for later genetic analysis. DNA from preserved tissues was extracted using the Qiagen DNeasy Blood and Tissue Kit (Qiagen Inc.) and rabbit species identified by PCR amplifying the D‐loop region of the mitochondrial DNA, and subsequently analyzing this region using two restriction fragment length polymorphism assays (Kovach et al., 2003; Litvaitis & Litvaitis, 1996; Litvaitis et al., 1997; Ryan et al., 2016). Microhematocrit tubes were centrifuged for 5 min within 8 hr of collection on a LW Scientific LWS‐M24 Microhematocrit Centrifuge machine at 14,800 g. Readings were taken visually and recorded to the nearest percent (Ryan et al., 2016).

Cottontails under 800 g were affixed with a 1.1‐g glue‐on radio transmitter with a winged mesh attachment following protocols outlined by Estes‐Zumpf and Rachlow (2007). Effort was made to recapture and reaffix transmitters to juveniles at regular intervals. Cottontails over 800 g were affixed with a 24‐g radio transmitter with a zip‐tie collar attachment (Advanced Telemetry Systems, Isanti, Minnesota). Transmitters were equipped with 8‐hr mortality switches. Following an adjustment period of 72 hr, cottontails were monitored weekly until a mortality event occurred, until the signal was lost, or at the termination of the study in April 2017. Any cottontails that died within the first 72 hr postcollaring were censored from analyses. To obtain fine‐scale habitat data, we tracked cottontails from December 2013 to October 2016 by locating individuals 1–2 times a week using triangulation. Additionally, we homed in on each individual once a week and recorded their location with a GPS unit as in Cheeseman et al. (2018). All work was conducted following guidelines set by the American Society of Mammalogists on use of wild mammals in research (Sikes & Animal Care & Use Committee of the American Society of Mammalogists, 2016) and with approval of the SUNY‐ESF IACUC, protocols #120801 and #151002.

We obtained estimates of leaf‐off season canopy closure, shrub height, and stem density of shrub species every 50 m in a grid across used portions of study sites as determined from telemetry data. Canopy closure during the leaf‐off season was estimated for the four cardinal directions at each sampling location with a densiometer held at a height of 1 m (Cheeseman et al., 2018). These estimates were averaged by sampling location to obtain the final canopy closure estimate. The number of woody stems less than 10 cm DBH and originating within a 10‐ × 1‐m plot was tallied by species and totaled to obtain total stem density of the plot. Stems were classified as native or invasive, and palatable native species were identified using Pringle (1960). Vegetation data were geolocated and rasterized using ArcMap 10.5.1 (ESRI), and interpolated to 10‐m^2^ intervals for comparison with telemetry locations, as described in Cheeseman et al. (2018).

### 2.3 Survival

Cottontails can be aged using hind foot length until they reach adult size and sexual maturity at around 4 months of age (Chapman, 1975; Chapman et al., 1980), after which point age of mature cottontails cannot be assessed noninvasively (Bothma et al., 1972). We therefore analyzed survival data at two age classes, postnestling juveniles and adults. Juveniles were available for capture after leaving their nest (i.e., around 14 days) and considered juveniles until their hind foot length was >80 mm and their weight was >800 g (Bothma et al., 1972; Chapman et al., 1980). As it was not consistently possible to recapture individuals to reassess weight and hind foot length, all remaining juveniles were considered adults 4 months after the end of the recorded breeding season (i.e., 1 December). Juvenile and adult survival were assessed separately using logistic exposure models, which allow for ragged entry, removal of individuals, and time‐varying covariates, and accommodate uneven intervals between observations (Shaffer, 2004). We used a sequential modeling approach and first developed three candidate sets of models with alternative environmental (seven adult and six juvenile models), health (14 adult and five juvenile models), and resource and landscape (23 adult and 19 juvenile models) parametrizations thought to influence survival of juvenile and adult cottontails (Table 1, Table A1; Arnold, 2010). We developed a combined candidate set of 13 adult and seven juvenile models including combinations of variables from the environmental, heath, and resource and landscape factor models with a ΔAIC_c_ value ≤ 2.0 (Table A1). We excluded correlated (*r* > 0.7) and nested variables (i.e., Japanese barberry stem counts are nested within total stems) from consideration in the same model. We considered the environmental model set to control for variability in survival due to weather, which has been hypothesized to impact survival of cottontails at northern latitudes (Bond et al., 2001; Weidman & Litvaitis, 2011). As we were interested in examining the difference between New England and eastern cottontails, “species” was included as a factor in all but the null models. Season has been found consistently an important predictor of lagomorph survival at northern latitudes (Keith & Bloomer, 1993; Trent & Rongstad, 1974; Weidman & Litvaitis, 2011). Therefore, we included season in all models of adult survival. As parturition occurs spring through late summer and most juveniles have matured to adults by the leaf‐off season, we did not include season in models for juvenile survival. Each model set was evaluated using an information theoretic approach and Akaike information criterion corrected for small sample size (AIC_c_; Burnham & Anderson, 2002). The final combined model set included combinations of variables within all supported weather, health, and landscape models and a null model. We performed model selection of the final candidate set using AIC_c_, and models with a ΔAIC_c_ ≤ 2.0 and with a corresponding reduction in deviance were considered to have support (Arnold, 2010). All analyses were conducted in Program R 3.5.3 (R Core Team, 2019). We used model predicted values to project survival probabilities of adult New England and eastern cottontails under several different hypothetical patch scenarios: early successional shrubland dominated by native plants, early successional shrubland dominated by invasive plants, late successional forest with low understory, late successional forest with invasive understory, and persistent forested shrubland.

**TABLE 1:** Covariates considered in three candidate logistic exposure model sets for assessing the effects of weather, individual health, and resource and landscape factors on survival of juvenile and adult New England cottontails (*Sylvilagus transitionalis*) and eastern cottontails (*Sylvilagus floridanus*) in New York, 2013–2017

Model set	Covariates	Description
Weather	Species	New England cottontail
		Eastern cottontail
	Season^a^	Leaf‐off (November–April)
		Leaf‐on (May–October)
	Snowfall^a^	Total precipitation in snow over past 7 days
	Maximum temperature^a^	Maximum temperature in past 7 days
	Snow depth^a^	Average snow depth over past 7 days
	Year^a^	Observation year 2013–2017
	Precipitation^b^	Average daily precipitation over past 7 days
	Minimum Temperature^b^	Minimum temperature in past 7 days
Health	Species	New England cottontail
		Eastern cottontail
	Season^a^	Leaf‐off (November–April)
		Leaf‐on (May–October)
	Body condition	Poor body condition at last capture
		Good body condition at last capture
	Hematocrit	Hematocrit reading at last capture
	Ticks	Number of ticks at last capture
	Weight^a^	Weight at last capture
Landscape	Species	New England cottontail
		Eastern cottontail
	Season^a^	Leaf‐off (November–April)
		Leaf‐on (May–October)
	Barberry	Average barberry stems 10 m^2^ at used locations per over past 28 days
	Palatable stems	Average native palatable stems per 10 m^2^ at used locations over past 28 days
	Canopy closure	Average canopy closure at used locations over past 28 days
	Distance	Average distance between locations over past 7 days
	Hunted^a^	Cottontail hunting permitted at site
	Total stems	Total stems per 10 m^2^ at used locations over past 28 days
	Patch area	Area of occupied shrubland
	Competition^a^	Low: eastern cottontails: New England cottontails < 1:6
		High: eastern cottontails: New England cottontails > 1:6

^a^ Only included in models of adult cottontails.

^b^ Only included in models of juvenile cottontails.

### 2.4 Capture per unit effort

Low recapture rates and dense habitat features prevented estimation of density using mark–recapture or distance survey methods so we estimated capture per unit effort (CPUE) as an indicator of local abundance (Barbour & Litvaitis, 1993). Our metric of CPUE was the number of new captures per trapping session. We modeled CPUE as a function of cottontail species and shrubland type, and included an index of competition for New England cottontails (i.e., interaction between New England cottontails, shrubland type, and the number of eastern cottontails known in the patch). Patches within sites were manually digitized, and shrubland type was identified as early, mid, late, or persistent based on canopy closure, shrub height, and dominant shrub communities inferred from aerial imagery, ground truthing, and vegetation surveys. We fit the model using a mixed‐effect zero‐inflated Poisson regression in the “glmmTMB” package in Program R, with site as the random effect because we had data from multiple sessions from most sites. No New England cottontails were captured in early successional patches where eastern cottontails were present, so the interaction between the early successional level of the shrubland‐type factor and eastern cottontail abundance was not included in the model. The number of trap nights and area trapped were included as offsets; thus, we predicted captures per 100 trap nights per ha.

## 3 RESULTS

### 3.1 Juvenile survival

**FIGURE 2:**
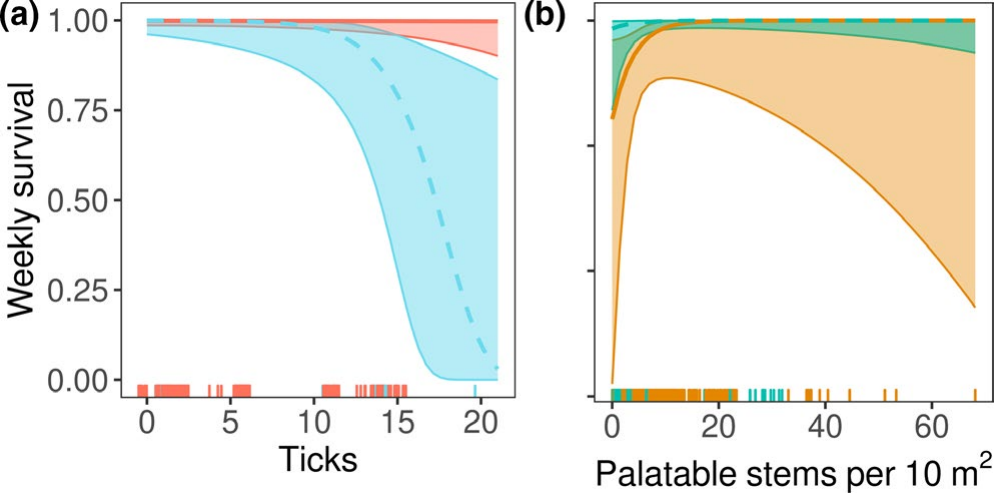
Predicted weekly survival and 95% prediction intervals of juvenile cottontails by a. tick burden given body condition is good (red, dark, solid line) or poor (blue, light, dashed line) and b. palatable stems per 10 m^2^ for New England cottontails (blue, light, solid line) and eastern cottontails (orange, dark, dashed line) in New York, 2013–2017, in good body condition. Trends in survival by ticks are shown for New England cottontails, eastern cottontails were similar and are not shown. Predictions made with other covariates held at their means. Observations shown in rugs

We monitored survival of 27 juvenile New England cottontails and 25 juvenile eastern cottontails. From these individuals, we observed 4 New England and 4 eastern cottontail mortalities from 345 and 315 observations, respectively. Species, body condition, tick burden, and palatable stem densities had support from both top combined models of juvenile survival (Table 2). However, the stem density effect and the main effect for body condition lacked power as a predictors in our dataset (Table 3). Survival of juvenile New England cottontails was higher than eastern cottontails (Table 3, predicted seasonal survival in Table A2). Tick burden had no effect on survival of juvenile rabbits in good body condition (Figure 2a), with weekly probability for survival of 1.00 (95% PI = 0.99–1.00) for New England cottontails and 0.99 (95% PI = 0.91–1.00) for eastern cottontails. However, for individuals in poor body condition, survival was strongly negatively correlated with tick burden (Figure 2a). Predicted weekly survival of juvenile cottontails approached 100% as long as palatable stem densities were >10 stems/10 m^2^, but the prediction intervals were very wide (Figure 2b). The second best model contained movement distance; however, the ΔAIC = 1.97 and there was only a minor reduction in deviance of 0.07, suggesting that the parameter movement distance had little explanatory power in the model. We found no evidence that weather variables affected juvenile survival (Table 2).

**TABLE 2:** Information theoretic model selection criteria for models of the effect of weather, health, and resource and landscape factors on juvenile and adult New England (*Sylvilagus transitionalis*) and eastern cottontail (*Sylvilagus floridanus*) survival in New York, 2013–2017

Age	Model set	Model	*K* ^a^	Relative likelihood^b^	AIC_c_ ^c^	ΔAIC_c_ ^d^	*w*_i_ ^e^	Deviance
Juvenile	Weather	Null	1	1.00	84.05	0.00	0.52	82.05
		Species	2	0.38	86.00	1.95	0.19	81.98
	Health	Species + ticks × body condition	5	1.00	70.92	0.00	0.98	60.82
		Null	1	0.00	84.05	0.00	0.00	82.05
	Landscape	Species + palatable stems	3	1.00	82.78	0.00	0.33	76.74
		Species + distance + palatable stems	4	0.62	83.72	0.95	0.20	75.66
		Null	1	0.53	84.05	1.28	0.17	82.05
	Combined	Species + ticks × body condition + palatable stems	6	1.00	68.13	0.00	0.62	56.00
		Species + ticks × body condition + distance + palatable stems	7	0.37	70.10	1.97	0.23	55.93
Adult	Weather	Species + leaf‐off + snow depth	4	1.00	1,279.72	0.00	0.47	1,271.72
		Species + leaf‐off + snowfall + snow depth	5	0.62	1,280.69	0.96	0.29	1,270.68
		Species + leaf‐off + year + snow depth	5	0.43	1,281.42	1.70	0.20	1,271.42
		Null	1	0.00	1,310.84	31.12	0.00	1,308.84
	Health	Species + leaf‐off × body condition	5	1.00	1,286.34	0.00	0.59	1,276.33
		Species + leaf‐off × body condition + tick season × ticks^f^	6	0.39	1,288.20	1.87	0.23	1,276.19
		Null	1	0.00	1,310.84	24.51	0.00	1,308.84
	Landscape	Species × canopy + leaf‐off + distance + barberry × palatable stems + canopy × barberry stems	10	1.00	1,280.16	0.00	0.55	1,260.14
		Null	1	0.00	1,310.84	30.68	0.00	1,308.84
	Combined	Species × canopy + leaf‐off × body condition + snow depth + distance + barberry × palatable stems + canopy × barberry stems	13	1.00	1,244.00	0.00	0.94	1,217.95
		Null	1	0.00	1,310.84	66.85	0.00	1,310.84

Note

Models with AIC_c_ < 2.0 and null weather, health, resource and landscape, and combined model sets. All models with interactions contained main effects unless otherwise noted.

^a^ *K* = number of parameters in the model.

^b^ Relative likelihood = exp (−0.5 × ΔAIC_c_), the likelihood ratio of the given model to the top model

^c^ AIC_c_ = AIC corrected for small sample sizes.

^d^ ΔAIC_c_ = difference in the AIC_c_ between a given model and the top model.

^e^ *w*_i_ = AIC_c_ weights, or the probability that of the models tested the given model fits the data best.

^f^ Ticks only included in models with the interaction of tick season.

**TABLE 3:** Regression parameter estimates for top ranked models of juvenile and adult New England (*Sylvilagus transitionalis*) and eastern cottontail (*Sylvilagus floridanus*) survival in New York, 2013–2017

Age	Rank	Parameter	Estimate	SE	95% LCB	95% UCB
Juvenile	1	Intercept	5.88	2.60	0.78	10.98
		Species	2.58	1.11	0.40	4.76
		Ticks	−0.49	0.20	−0.88	−0.10
		Body condition	−1.94	2.66	−7.15	3.27
		Palatable stems	27.11	15.73	−3.72	57.94
		Ticks × body condition	0.44	0.22	0.01	0.87
	2	Intercept	6.05	2.64	0.88	11.22
		Species	2.57	1.11	0.39	4.75
		Ticks	−0.50	0.20	−0.89	−0.11
		Body condition	−1.94	2.63	−7.09	3.21
		Distance	−0.29	1.04	−2.33	1.75
		Palatable stems	27.50	16.15	−4.15	59.15
Adult	1	Intercept	3.89	0.56	2.79	4.99
		Leaf‐off	1.44	0.60	0.26	2.62
		Body condition	2.27	0.41	1.47	3.07
		Species	−0.42	0.51	−1.42	0.58
		Snow	−3.55	0.70	−4.92	−2.18
		Distance	−0.12	0.03	−0.18	−0.06
		Barberry	−1.29	1.39	−4.01	1.43
		Palatable stems	6.20	2.11	2.06	10.34
		Canopy	−1.05	0.87	−2.76	0.66
		Leaf‐off × body condition	−2.30	0.63	−3.53	−1.07
		Barberry × palatable stems	−10.10	5.02	−19.94	−0.26
		Species × canopy	1.62	1.07	−0.48	3.72
		Barberry × canopy	4.41	2.40	−0.29	9.11

Note

Regression parameter estimates and their standard errors and 95% lower and upper confidence bounds shown for the top combined models. Rankings determined from ΔAIC_c_ scores.

### 3.2 Adult survival

We monitored survival of 82 adult New England cottontails and 76 adult eastern cottontails. From these individuals, we observed 59 mortalities from 3,829 observations of New England cottontails and 69 mortalities from 4,753 observations of eastern cottontails. Survival of both New England and eastern cottontails was influenced by weather, health, and landscape features, but did not appear to vary by levels of interspecific competition, which was not supported in any top model (Table 2).

**FIGURE 3:**
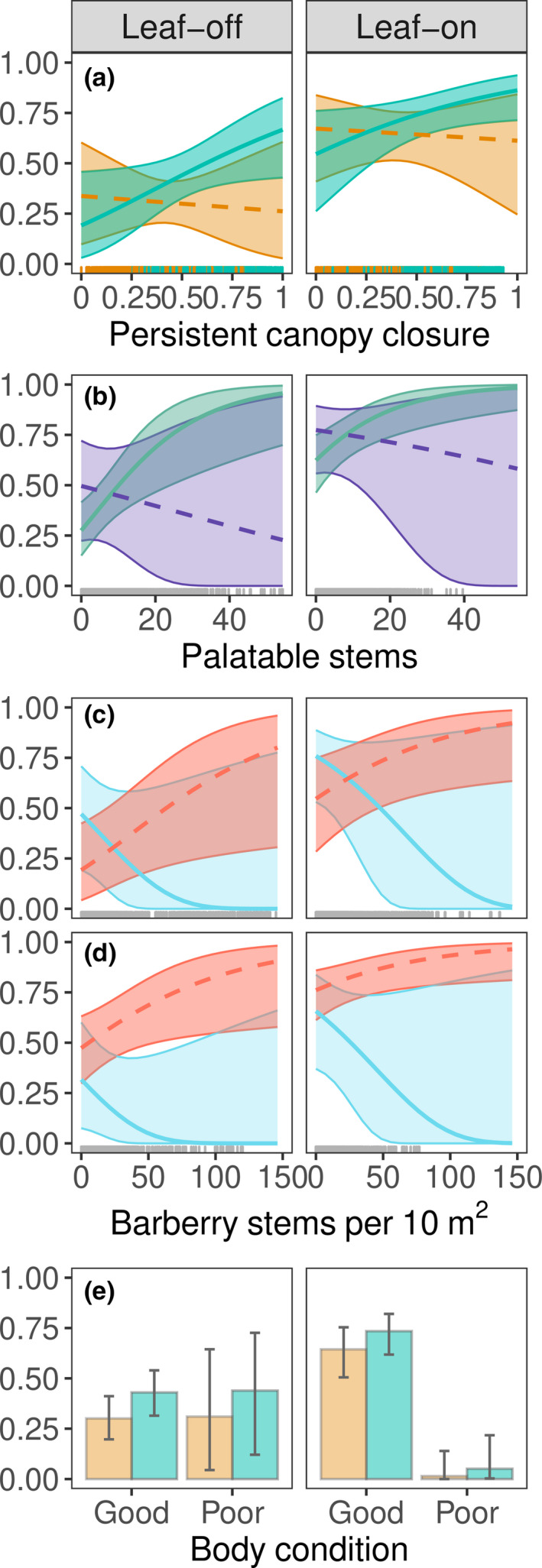
Predicted seasonal survival and 95% prediction intervals for adult cottontails in the leaf‐off (November–April) and leaf‐on (May–October) seasons by (a) canopy closure of the area used over the past 28 days (New England cottontails, blue, light, solid line; eastern cottontails, orange, orange, dark, dashed line) and (b) palatable stem densities where Japanese barberry stems are dense (75 stems per 10 m^2^, purple, dark, dashed line) and where Japanese barberry stems are sparse (0 stems per 10 m^2^, green, solid, light line) in the area used over the past 28 days; Japanese barberry stem densities at levels of canopy closure that are high (100%, red, dark, dashed line) and low (0%, blue, light, solid line) for (c) eastern cottontails and (d) New England cottontails, and (e) body condition (New England cottontails, blue, light, solid line; eastern cottontails, orange, orange, dark, dashed line). Where species‐specific interactions with variables were not supported by top models, predictions are shown for New England cottontails and for cottontails in good body condition unless otherwise noted. All predictions were made with other model variables held at their means. Observations from New York 2013–2017 shown in rugs

Based on overlap of prediction intervals (PI), annual probability of survival for individuals in good condition and with covariates held at their seasonal means was not different between New England cottontails (x̄ = 0.32, 95% PI = 0.19–0.44) and eastern cottontails (x̄ = 0.19, 95% PI = 0.10–0.31; predicted seasonal survival in Table A2). Although all canopy closure terms in the regression model had low predictive power (Table 3), a trend toward increased survival of adult New England cottontails with increasing canopy closure was evident (Figure 3a). Eastern cottontail survival did not depend on canopy closure (Figure 3a); however, there were no observations of eastern cottontails at the highest levels of canopy closure. Survival increased with increasing palatable stem density for both species, but this effect was only apparent where the common invasive shrub Japanese barberry was not prevalent (Figure 3b). Survival of both eastern cottontails (Figure 3c) and New England cottontails (Figure 3d) showed a positive correlation with Japanese barberry stem density where tree canopy closure was high, though the predictive power of this variable was low. We did not detect an effect of Japanese barberry where canopy closure was low for either eastern cottontails (Figure 3c) or New England cottontails (Figure 3d).

Body condition was also an important predictor of survival for adults of both species, with the effect depending on season (Table 2). New England and eastern cottontails in good body condition had lower survival during the leaf‐off season than in the leaf‐on season (Figure 3e). Individuals in poor body condition in the leaf‐on season had lower survival than individuals in any other combination of body condition and season (Figure 3e).

**FIGURE 4:**
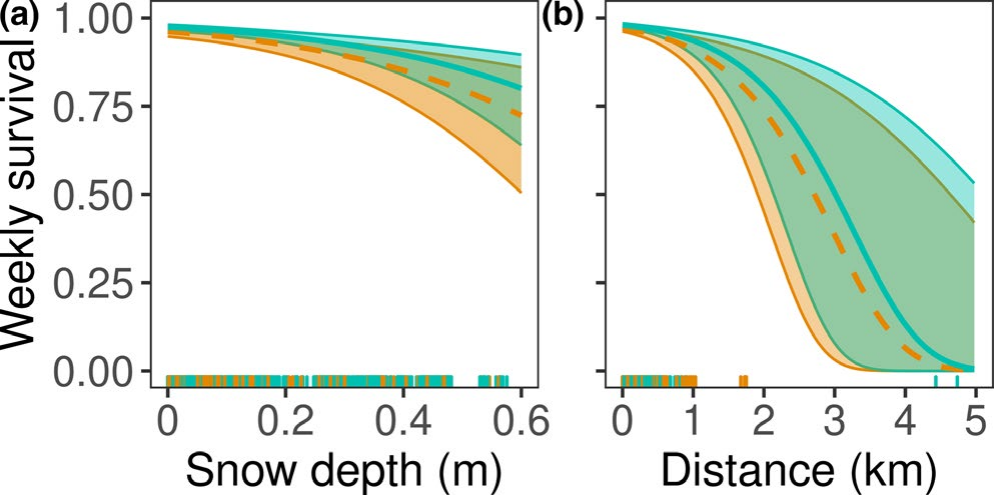
Impacts of snow depth and movement distance on weekly survival of adult New England cottontails (*Sylvilagus transitionalis,* blue, light, solid line) and eastern cottontails (*Sylvilagus floridanus,* orange, dark, dashed line) in New York, 2013–2017. Predicted weekly survival and 95% prediction intervals shown by a. snow depth (leaf‐off, November–April) season only; and b. distance moved over the last 7 days. Predictions between season and species for distance were similar and are not shown. All predictions were made with other model variables held at their means. Observations shown in rugs

Survival was negatively correlated with movement and snow depth and did not differ between species (Table 3). Survival during the leaf‐off season was generally high under no snow conditions approaching a weekly probability of 0.97 (95% PI: 0.96–0.98) for New England cottontails, but the weekly probability of survival was only 0.82 (95% PI: 0.69–0.90) for New England cottontails at the maximum recorded snow depth of 56 cm (Figure 4a). For movement distance, predicted weekly survival for New England cottontails was 0.96 (95% PI: 0.95–0.97) when average movement distance in the previous week was 50 m, but when movement distance was 2 km, such as might occur during dispersal, weekly probability of survival was 0.80 (95% PI: 0.57–0.92; Figure 4b).

**FIGURE 5:**
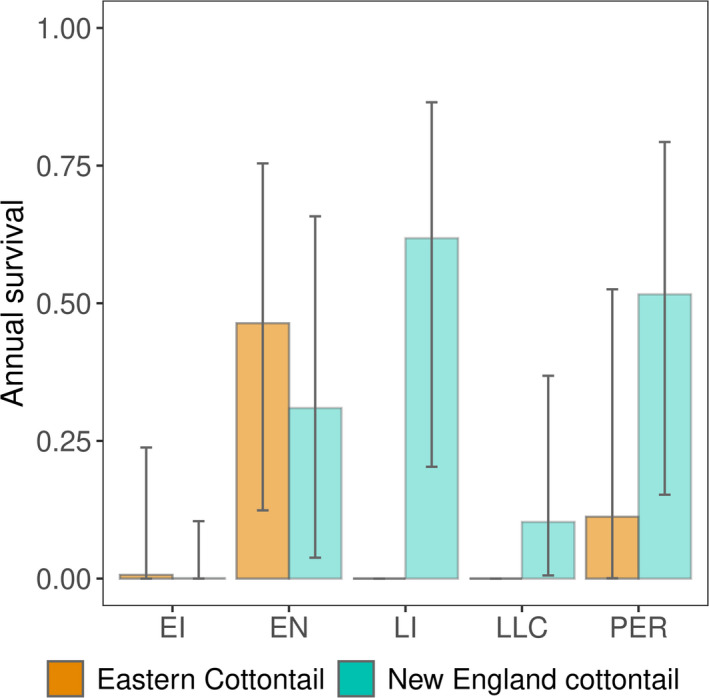
Survival of New England cottontail (*Sylvilagus transitionalis*) and eastern cottontail (*Sylvilagus floridanus*) in hypothetical shrublands. Predicted annual survival and 95% prediction intervals shown for hypothetical shrublands based on typical habitat metrics in New York, 2013–2017. Shrubland types include early invasive (EI, 0% tree canopy, 50 stems/10 m^2^ Japanese barberry, 0 stems/10 m^2^ native palatable shrubs), early native (EN, 0% tree canopy, 0 stems/10 m^2^ Japanese barberry, 20 stems/10 m^2^ native palatable shrubs), late invasive (LI, 100% tree canopy, 50 stems/10 m^2^ Japanese barberry, 0 stems/10 m^2^ native palatable shrubs), late low shrub cover (LLC, 0% tree canopy, 0 stems/10 m^2^ Japanese barberry, 0 stems/10 m^2^ native palatable shrubs), and persistent (PER, 100% tree canopy, 0 stems/10 m^2^ Japanese barberry, 20 stems/10 m^2^ native palatable shrubs)

Predicted cottontail survival in hypothetical shrublands varied by patch type and species. Survival of New England cottontails was lower in invasive‐dominated early successional shrublands and patches with full tree canopy but low shrub understory than other patch types and only nonzero for eastern cottontails within early and mid‐successional and persistent shrublands (Figure 5). Although no other comparisons were significantly different, the results suggest that in general, New England cottontail survival was lower in early successional shrubland than late successional and persistent shrublands (Figure 5).

### 3.3 Captures per unit effort

**FIGURE 6:**
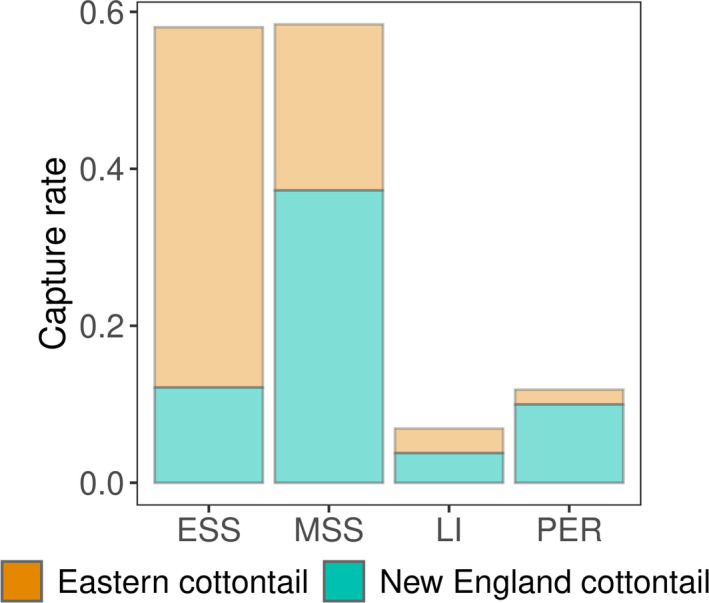
Densities of New England cottontails (*Sylvilagus transitionalis, bottom*) and eastern cottontails (*Sylvilagus floridanus, top*) by shrubland type in New York 2013–2016. Predictions for captures per 100 trap nights per ha shown for early successional shrubland (ESS), mid‐successional shrubland (MSS), late successional shrubland (LI), and persistent shrubland (PER). For New England cottontails, predictions are given for the mean numbers of eastern cottontails known to be present

**FIGURE 7:**
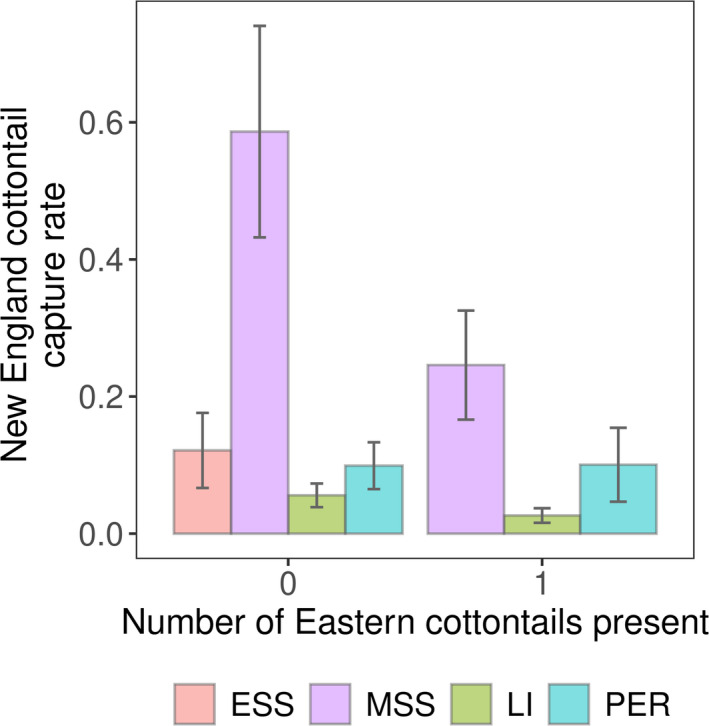
Impacts of eastern cottontail (*Sylvilagus floridanus*) abundance on New England cottontail (*Sylvilagus transitionalis*) density in New York, 2013–2016. Predicted captures per 100 trap nights per hectare and standard errors from left to right at each value of number of eastern cottontails known alive on the patch: early successional shrubland (ESS, not shown for 1 eastern cottontail present as there were no obseravtions in theis category), mid‐successional shrubland (MSS), invasive‐dominated late successional shrubland (LI), and native‐dominated persistent successional shrubland (PER). Only a single New England cottontail was captured during a session where two eastern cottontails were present in a patch (mid‐successional shrubland, not shown)

Our dataset included 69 captures of eastern cottontails and 112 captures of New England cottontails in 30,069 trap nights distributed across 66 patches in 15 sites. Shrubland type had a strong effect on CPUE of both New England and eastern cottontails (Table 4). Eastern cottontail CPUE was higher in early and mid‐successional shrublands than late successional and persistent shrublands (Figure 6). Given the mean number of eastern cottontails known alive, New England cottontail CPUE was greater in mid‐successional shrubland than any other shrubland type (Figure 6). However, in all shrubland types except persistent shrubland the CPUE of New England cottontails decreased with increasing eastern cottontails, such that with one eastern cottontail present, there was no difference between CPUE of mid‐successional and persistent shrublands (Figure 7). In fact, CPUE for New England cottontails in early successional shrubland was only nonzero in patches where there were no known eastern cottontails (Figure 7). Only a single New England cottontail was captured during a session where two eastern cottontails were known present in a patch (mid‐successional shrubland).

**TABLE 4:** Regression parameter estimates for a model of New England cottontail (*Sylvilagus transitionalis*) and eastern cottontail (*Sylvilagus floridanus*) density in New York, 2013–2016

Parameter	Estimate	*SE*	95% LCB	95% UCB
Intercept (eastern cottontail, mid‐successional)	−15.063	0.311	−15.672	−14.454
New England cottontail	1.020	0.309	0.413	1.626
Early	0.775	0.369	0.052	1.498
Late	−1.913	0.443	−2.782	−1.044
Persistent	−2.421	0.576	−3.550	−1.292
New England cottontail × early	−2.350	0.557	−3.441	−1.259
New England cottontail × late	−0.438	0.526	−1.468	0.592
New England cottontail × persistent	0.643	0.620	−0.571	1.857
New England cottontail × eastern cottontail abundance × mid	−0.869	0.309	−1.476	−0.263
New England cottontail × eastern cottontail abundance × late	−0.748	0.446	−1.622	0.126
New England cottontail × eastern cottontail abundance × persistent	0.014	0.536	−1.037	1.065

Note

Regression parameter estimates and their standard errors and upper (95% UCB) and lower (95% LCB) 95% confidence bounds shown.

## 4 DISCUSSION

For New England cottontails and many other imperiled species that are data‐limited, conservation has often proceeded based on the standard of “best available science” (Doremus, 2004). In such cases, it is common for knowledge from one part of a species’ range to be applied to conservation planning in a less‐studied location, or ecological theory may be leveraged to make broader inferences from correlative studies (Murphy & Weiland, 2014). These practices rest on the assumption of generalizability of results among studies and of ecological theory to natural systems. However, conservation management is usually applied in manipulated or disturbed systems, sometimes in remnants of intact habitat, or as part of habitat restoration efforts. Habitat quality varies across time and space and can generate deviations from selection under an ideal free distribution—where such deviations occur, the application of “best available science” to species management can result in the expenditure of considerable effort to create maladaptive habitat. For New England cottontail, we found habitat use is inconsistent with selection following an ideal free distribution. Both survival and catch per unit effort of New England cottontails were low in early successional shrublands shown to be selected over other shrubland types, while survival and CPUE for eastern cottontails were greatest under conditions representative of early successional shrublands (Cheeseman et al., 2018). Further, eastern cottontails reduced the CPUE of New England cottontails within early and mid‐successional shrublands, resulting in complex trade‐offs in habitat quality based on eastern cottontail abundance and shrubland age. Given these findings, habitat management goals targeting early and mid‐successional shrublands may be ineffective where eastern cottontail abundance is high. In this scenario, the creation of open, early successional shrublands may be inadvertently promoting non‐native eastern cottontail, while creating an ecological trap for the imperiled New England cottontail.

Early successional shrublands contain resources such as woody and herbaceous forage and cover, which are thought to be attractive to New England cottontails (Barbour & Litvaitis, 1993; Cheeseman et al., 2018). As a result, management to increase New England cottontail populations has primarily focused on creating shrublands with high stem densities and is often achieved by clearcutting, mowing, or burning to reset succession. However, such anthropogenic practices that create large patches of early successional shrubland may not appropriately mimic natural historic processes (i.e., shrublands resulting from beaver activity, blow downs, ice storms, or forest fires; Litvaitis, 2001). As a result, anthropogenically created shrublands may result in novel ecosystems for northeastern shrubland species, particularly given the presence and abundance of exotic invasive shrubs within modern successional shrublands (Johnson et al., 2006).

For cottontails, density may be negatively impacted by invasive shrubs in late successional shrublands and contribute to lower New England cottontail survival within younger shrublands. Maladaptive selection for the architecture and leaf phenology of invasive shrubs is a driver of ecological traps for several understory nesting birds (Battin, 2004). It is possible that New England cottontails are using similarly inappropriate cues to evaluate habitat quality in this novel context, resulting in maladaptive selection for these areas. However, invasive exotic shrubs, such as Japanese barberry, also support higher tick abundances than areas of low or native shrub cover (Elias et al., 2006; Lubelczyk et al., 2004; Williams et al., 2009) and have been linked to increased tick burdens on New England cottontails when compared to native shrub‐dominated habitats (Mello, 2018). High tick burdens are also associated with lower fecundity (Keith & Cary, 1990), greater morbidity (Scott et al., 2014), and greater mortality (Jones et al., 2018) in wildlife populations, and have been implicated in population declines of species including moose (DeIgiudice et al., 1997), several African ungulates (Lightfoot & Norval, 1981), and cottontails (Smith & Cheatum, 1944). For New England and eastern cottontails, ticks were an important predictor of juvenile survival, with even moderate tick burdens resulting in high survival costs for individuals in poor body condition. Notably, survival of juveniles in poor body condition with low tick burdens was not different from juveniles in good body condition, suggesting tick burden may be directly impacting survival for individuals that are already in poor health. As such, we suggest that tick abundance may reduce the quality of invasive shrub‐dominated habitats. We further note that while tick burden was not implicated in models of adult survival, individuals in poor body condition had reduced survival during the leaf‐on season when ticks are active. Baselines for juvenile health used in survival analyses were typically obtained within a month of transmitter loss, failure, or observed mortality, whereas we were unable to regularly recapture adult cottontails. Thus, the reduction in leaf‐on season survival for adults in poor condition could have been related to tick burdens, but tick burden at last capture is a poor indicator of tick burden at mortality.

Moreover, management practices targeting native early successional shrublands may more similarly mimic natural ecosystems in the Midwest and Great Plains regions, which are among the historic source populations for introduced eastern cottontails (Chapman & Morgan, 1973). Consistent with an evolutionary adaptation for early successional shrublands, Smith and Litvaitis (2000) found eastern cottontails better exploited resources and avoided predation within low cover areas than New England cottontails. As a result, eastern cottontails likely have an advantage in low cover patches, which could explain the species‐specific trends in survival in early successional shrublands. Regardless, early successional shrublands appear to provide high‐quality habitat to eastern cottontails where they are able to exclude New England cottontails (Cheeseman et al., 2018; Probert & Litvaitis, 1996).

In this study, CPUE for New England cottontails was greatest within mid‐successional shrublands, while point estimates for survival in mid‐successional shrublands were intermediate of early and late successional shrublands. It is possible that higher cottontail density within early successional shrublands compensates for the lower survival of New England cottontails in that cover type when compared to late successional and persistent shrublands through higher reproductive rates, resulting in a trade‐off between risky high‐productivity early successional shrubland and safe low‐productivity late successional and persistent shrublands. However, this trade‐off would be imbalanced by reduced New England cottontail density where eastern cottontails are present, resulting in sink habitat or an ecological trap induced by competitive interactions.

We do not know the exact mechanisms for increased survival of New England cottontails with shrubland age. One hypothesis is density‐dependent predation, which has been well documented in leporids (Krebs et al., 1995). Overall cottontail density was highest in early and mid‐successional shrublands, likely a result of abundant forage and cover in early and mid‐successional shrublands. However, higher density in early and mid‐successional shrublands could result in higher rates of density‐dependent predation than in late successional and persistent shrublands. Further, New England cottontails appear less well adapted to avoiding predation than eastern cottontails and incur higher survival costs in less structurally complex habitat (Smith & Litvaitis, 1999, 2000). As a result, New England cottontails may have greater predation risk in co‐occupied patches than eastern cottontails.

High dispersal costs and reduced movement capacity limit the ability for species to distribute among habitats in response to density (Stamps et al., 2005). Movement distance had a high survival cost for both species. Studies examining dispersal of New England cottontails have suggested a high rate of exploratory movement, but low rates of successful dispersal, possibly due to scarcity of suitable habitat within dispersal distance (Cheeseman, 2017). These conclusions are supported by levels of fragmentation and movement observed in population genetic studies (Bauer, 2018; Cheeseman et al., 2019; Fenderson et al., 2014). With high cost of movement and high search cost, New England cottontails may face high rates of diminishing fitness as population densities increase and they are unable to freely disperse across the landscape. While the impacts of limited dispersal at the metapopulation scale are widely recognized, high‐risk exploratory and dispersal movements could also serve to reduce population viability at the patch level by reducing survival below sustainable levels. Reducing the distance between suitable patches through placement of newly created habitat or the creation of stepping‐stone patches or corridors may improve survival of dispersing individuals at both patch and metapopulation scales.

In summary, we caution that by focusing management and restoration efforts on apparently attractive, but low‐quality habitat, the conservation strategy for New England cottontails may be fostering patches that promote eastern cottontails but act as ecological traps for New England cottontail. Early successional shrublands have been a target of New England cottontail conservation over the past decade, at considerable expense to regional wildlife management agencies and federal partners. Management actions should consider the trade‐offs between survival, density, shrubland, age, and presence of eastern cottontails when planning habitat restoration for the New England cottontail. In general, early successional shrublands are unlikely to provide high‐quality habitat, particularly where invasive shrubs or eastern cottontails are prevalent, while the suitability of mid‐successional habitat will depend on the suitability of the stand for eastern cottontails and their local presence. Older and persistent shrublands with suitable understory densities may provide adequate source habitat in the presence of eastern cottontail and should be a focus of habitat management efforts where New England and eastern cottontails are sympatric. However, effective conservation will be a challenge without further understanding the relative roles of different‐aged forest stands in cottontail metapopulation dynamics. We recommend that habitat management plans for New England cottontails move away from practices that generate early successional shrublands, where possible, and that strategies balance canopy retention and native shrub regeneration with the goal of maximizing both canopy closure and native shrub density. We further note that management to reduce invasive shrubs within shrublands without providing alternative cover will result in the destruction of habitat, or risk creation of sink habitat for New England cottontails.

Conservation of wildlife species and their habitat must necessarily be undertaken with incomplete knowledge of system dynamics. However, in doing so, we risk enacting management strategies that are ineffective or even detrimental to management goals. Uncertainty is often high when ecosystems are anthropogenically disturbed, and management must account for multiple interacting variables. In the case of the New England cottontail, fitness, density, and habitat selection are decoupled in highly impacted environments, leading to inaccurate inferences of habitat quality from previous habitat selection studies or when extrapolated from studies in less impacted areas. We demonstrate how complex interactions can alter habitat quality and result in wide‐scale implementation of potentially maladaptive management strategies. It is imperative that population responses to conservation based on such assumptions be monitored and evaluated regularly, and that ideally, management in the face of uncertainty proceed adaptively (Murphy & Weiland, 2016).

## CONFLICT OF INTEREST

The authors declare no conflicts of interest.

## AUTHOR CONTRIBUTIONS

**Amanda E. Cheeseman:** Conceptualization (lead); data curation (lead); formal analysis (lead); funding acquisition (equal); investigation (equal); methodology (lead); project administration (lead); visualization (lead); writing – original draft (lead); writing – review and editing (lead). **Jonathan B. Cohen:** Conceptualization (equal); data curation (supporting); formal analysis (supporting); funding acquisition (equal); investigation (supporting); methodology (supporting); project administration (equal); supervision (lead); writing – original draft (supporting); writing – review and editing (supporting). **Sadie Ryan:** Conceptualization (supporting); data curation (supporting); funding acquisition (equal); investigation (supporting); methodology (supporting); project administration (supporting); writing – review and editing (equal). **Christopher M. Whipps:** Conceptualization (supporting); data curation (supporting); funding acquisition (equal); investigation (supporting); methodology (supporting); project administration (supporting); writing – review and editing (equal).

## Supporting information

Appendix S1 and S2

## Data Availability

Data are available from the Dryad Digital Repository at https://doi.org/10.5061/dryad.3bk3j9khq.

## Appendix

**TABLE A1:** Impacts of weather, health, and resource and landscape factors on juvenile and adult New England (*Sylvilagus transitionalis*) and eastern cottontail (*Sylvilagus floridanus*) survival in New York, 2013–2017

Age	Set	Model	*K* ^a^	Relative likelihood^b^	AICc^c^	ΔAIC_c_ ^d^	*w*_i_ ^e^	Deviance
Juvenile	Weather	Null	1	1.00	84.05	0.00	0.52	82.05
		Species	2	0.38	86.00	1.95	0.19	81.98
		Species + precipitation	3	0.19	87.35	3.29	0.10	81.31
		Species + minimum temperature	3	0.18	87.44	3.38	0.10	81.40
		Species + year	3	0.14	87.99	3.94	0.07	81.96
		Species + precipitation × minimum temperature	5	0.05	90.19	6.14	0.02	80.10
	Body	Species + ticks × body condition	5	1.00	70.92	0.00	0.98	60.82
		Species + ticks	3	0.01	79.88	8.96	0.01	73.84
		Species + body condition	3	0.01	80.66	9.75	0.01	74.62
		Null	1	0.00	84.05	13.14	0.00	82.05
		Species + hematocrit	3	0.00	86.92	16.01	0.00	80.89
	Landscape	Species + palatable stems	3	1.00	82.78	0.00	0.33	76.74
		Species + movement distance + palatable stems	4	0.62	83.72	0.95	0.20	75.66
		Null	1	0.53	84.05	1.28	0.17	82.05
		Species	2	0.20	86.00	3.23	0.07	81.98
		Species + movement distance + barberry stems × palatable stems	6	0.10	87.37	4.60	0.03	75.25
		Species + movement distance	3	0.09	87.52	4.74	0.03	81.48
		Species + patch area	3	0.09	87.67	4.89	0.03	81.63
		Species + barberry stems	3	0.08	87.81	5.03	0.03	81.77
		Species × competition^f^ + Species	3	0.07	88.00	5.22	0.02	81.96
		Species + canopy	3	0.07	88.01	5.24	0.02	81.98
		Species × patch area	4	0.05	88.83	6.05	0.02	80.77
		Species + barberry stems + movement distance	4	0.04	89.37	6.59	0.01	81.30
		Species + barberry stems × canopy	5	0.03	90.14	7.36	0.01	80.05
		Species + barberry stems + movement distance + patch area	5	0.02	91.10	8.32	0.01	81.00
		Species + canopy + movement distance + patch area	5	0.01	91.23	8.45	0.01	81.14
		Species + barberry stems + canopy + movement distance	5	0.01	91.35	8.58	0.01	81.26
		Barberry stems + canopy × species	5	0.01	91.39	8.62	0.00	81.30
		Species + patch area × total stems	5	0.01	91.55	8.77	0.00	81.46
		Total stems × canopy + species	5	0.01	91.92	9.15	0.00	81.83
	Combined	Species + ticks × body condition + palatable stems	6	1.00	68.13	0.00	0.62	56.00
		Species + ticks × body condition + movement distance + palatable stems	7	0.37	70.10	1.97	0.23	55.93
		Species + ticks × body condition	5	0.25	70.92	2.79	0.15	60.82
		Null	1	0.00	84.05	15.93	0.00	82.05
		Species	2	0.00	86.00	17.88	0.00	81.98
Adult	Weather	Species + snow depth + leaf‐off	4	1.00	1,279.72	0.00	0.47	1,271.72
		Species + snow fall + snow depth + leaf‐off	5	0.62	1,280.69	0.96	0.29	1,270.68
		Species + year + leaf‐off + snow depth	5	0.43	1,281.42	1.70	0.20	1,271.41
		Species + snow fall × maximum temperature + snow depth + leaf‐off	7	0.09	1,284.58	4.85	0.04	1,270.56
		Species + leaf‐off	3	0.00	1,297.01	17.28	0.00	1,291.00
		Species + leaf‐off + year	4	0.00	1,298.91	19.18	0.00	1,290.90
		Null	1	0.00	1,310.84	31.12	0.00	1,308.84
	Body	Species + leaf‐off × body condition	5	1.00	1,286.34	0.00	0.59	1,276.33
		Species + leaf‐off × body condition + tick season × ticks^f^	6	0.39	1,288.20	1.87	0.23	1,276.19
		Leaf‐off × body condition + species + tick season × ticks^f^ × body condition	7	0.15	1,290.19	3.86	0.09	1,276.18
		Species + leaf‐off + body condition	4	0.07	1,291.80	5.46	0.04	1,283.79
		Species + leaf‐off + tick season × ticks^f^ × body condition	6	0.04	1,292.94	6.60	0.02	1,280.93
		Species + leaf‐off + tick season × ticks^f^ + weight + body condition	6	0.03	1,293.24	6.90	0.02	1,281.23
		Species + leaf‐off + tick season × ticks^f^ + weight + body condition + hematocrit	7	0.01	1,295.17	8.84	0.01	1,281.16
		Species + leaf‐off + weight	4	0.01	1,296.45	10.12	0.00	1,288.45
		Species + leaf‐off	3	0.00	1,297.01	10.67	0.00	1,291.00
		Species + leaf‐off + tick season × ticks^f^ × weight	6	0.00	1,298.50	12.17	0.00	1,286.49
		Species + leaf‐off + tick season × ticks^f^	4	0.00	1,298.73	12.39	0.00	1,290.72
		Species + leaf‐off + hematocrit	4	0.00	1,298.76	12.42	0.00	1,290.75
		Species + leaf‐off + tick season × ticks^f^ × hematocrit	6	0.00	1,302.56	16.22	0.00	1,290.55
		Null	1	0.00	1,310.84	24.51	0.00	1,308.84
	Landscape	Movement distance + barberry stems × palatable stems + leaf‐off + canopy × species + canopy × barberry stems	10	1.00	1,280.16	0.00	0.55	1,260.14
		Movement distance + barberry stems × palatable stems + leaf‐off + canopy × species + canopy	9	0.35	1,282.29	2.13	0.19	1,264.27
		Leaf‐off × species × competition^f^ + movement distance + barberry stems × palatable stems + leaf‐off + canopy × Species + canopy × barberry stems	12	0.25	1,282.92	2.75	0.14	1,258.88
		Movement distance + leaf‐off × species × competition^f^ + leaf‐off + canopy × species + canopy × barberry stems	10	0.10	1,284.72	4.56	0.06	1,264.70
		Leaf‐off × species × competition^f^ + patch area + movement distance + hunt	7	0.04	1,286.40	6.24	0.02	1,272.39
		Leaf‐off × species × competition^f^ + patch area + movement distance + hunt + barberry stems × palatable stems + leaf‐off + canopy × species + canopy × barberry stems	14	0.04	1,286.59	6.42	0.02	1,258.54
		Leaf‐off × species × competition^f^ + movement distance + leaf‐off × canopy × species + total stems × canopy	13	0.02	1,288.35	8.19	0.01	1,262.31
		Movement distance + patch area + leaf‐off × species × competition^f^ + hunt + barberry stems × palatable stems + leaf‐off + canopy × species + canopy	13	0.02	1,288.52	8.36	0.01	1,262.48
		Movement distance + leaf‐off × canopy × species × total stems	17	0.01	1,290.46	10.30	0.00	1,256.39
		Leaf‐off × species × competition^f^ + patch area + movement distance + hunt + leaf‐off × canopy × species × total stems	21	0.00	1,296.05	15.88	0.00	1,253.94
		Species + leaf‐off	3	0.00	1,297.01	16.84	0.00	1,291.00
		Barberry stems × palatable stems + leaf‐off + species + canopy × species + canopy × barberry stems	9	0.00	1,297.44	17.28	0.00	1,279.42
		Patch area + barberry stems × palatable stems + leaf‐off + species + canopy × species + canopy × barberry stems	10	0.00	1,299.18	19.02	0.00	1,279.15
		Patch area + leaf‐off + canopy × species + canopy × barberry stems	8	0.00	1,299.74	19.58	0.00	1,283.73
		Leaf‐off × species × competition^f^ + barberry stems × palatable stems + leaf‐off + canopy × species + canopy × barberry stems	11	0.00	1,300.01	19.85	0.00	1,277.98
		Leaf‐off × species × competition^f^ + patch area + barberry stems × palatable stems + leaf‐off + canopy × species + canopy × barberry stems	12	0.00	1,301.78	21.61	0.00	1,277.74
		Leaf‐off × canopy × species + total stems × canopy	10	0.00	1,302.26	22.10	0.00	1,282.23
		Patch area + leaf‐off × species × competition^f^ + barberry stems × palatable stems + leaf‐off + canopy × species	11	0.00	1,302.96	22.80	0.00	1,280.93
		Leaf‐off × species × competition + patch area + leaf‐off × canopy × species + total stems × canopy	13	0.00	1,305.45	25.29	0.00	1,279.41
		Leaf‐off × canopy × species × total stems	16	0.00	1,307.04	26.87	0.00	1,274.97
		Patch area + leaf‐off × canopy × species × total stems	17	0.00	1,307.85	27.69	0.00	1,273.78
		Hunt + leaf‐off × canopy × species × total stems	17	0.00	1,308.41	28.25	0.00	1,274.34
		Leaf‐off × species × competition + leaf‐off × canopy × species × total stems	18	0.00	1,309.38	29.22	0.00	1,273.30
		Null	1	0.00	1,310.84	30.68	0.00	1,308.84
	Combined	Leaf‐off × body condition + snow depth + movement distance + barberry stems × palatable stems + canopy × species + canopy × barberry stems	13	0.70	1,244.00	0.70	0.70	1,217.95
		Leaf‐off × body condition + snow depth + year + snow fall + movement distance + barberry stems × palatable stems + canopy × species + canopy × barberry stems	15	0.31	1,246.34	2.35	0.22	1,216.29
		Leaf‐off × body condition + tick season × ticks^f^ + snow depth + year + snow fall + movement distance + barberry stems × palatable stems + leaf‐off + canopy × species + canopy × barberry stems	16	0.11	1,248.35	4.35	0.08	1,216.29
		Leaf‐off × body condition + tick season × ticks + year + snow fall + movement distance + barberry stems × palatable stems + leaf‐off + canopy × species + canopy × barberry stems	15	0.00	1,258.25	14.26	0.00	1,228.20
		Snow depth + movement distance + barberry stems × palatable stems + leaf‐off + canopy × species + canopy × barberry stems	11	0.00	1,261.39	17.40	0.00	1,239.36
		Snow depth + snow fall + movement distance + barberry stems × palatable stems + leaf‐off + canopy × species + canopy × barberry stems	12	0.00	1,262.19	18.20	0.00	1,238.16
		Snow depth + snow fall + tick season × ticks^f^ + movement distance + barberry stems × palatable stems + leaf‐off + canopy × species + canopy × barberry stems	13	0.00	1,263.85	19.85	0.00	1,237.81
		Leaf‐off × body condition + tick season × ticks + species + snow depth + year + snow fall × maximum temperature	11	0.00	1,276.83	32.84	0.00	1,254.80
		Movement distance + barberry stems × palatable stems + leaf‐off + canopy × species + canopy × barberry stems	10	0.00	1,280.16	36.17	0.00	1,260.14
		Species + snow depth + leaf‐off + year + snow fall	6	0.00	1,282.42	38.43	0.00	1,270.41
		Leaf‐off × body condition + species + tick season × ticks^f^	6	0.00	1,288.20	44.21	0.00	1,276.19
		Species + leaf‐off	3	0.00	1,297.01	53.01	0.00	1,291.00
		Null	1	0.00	1,310.84	66.85	0.00	1,308.84

Note

All considered models shown for weather, health, resource and landscape, and combined model sets. All models with interactions contained main effects unless otherwise noted.

^a^ *K* = number of parameters in the model.

^b^ Relative Likelihood = exp (−0.5 × ΔAIC_c_), the likelihood ratio of the given model to the top model.

^c^ AIC_c_ = AIC corrected for small sample sizes.

^d^ ΔAIC_c_ = difference in the AIC_c_ between a given model and the top model.

^e^ *w*_i_ = AIC_c_ weights, or the probability that of the models tested the given model fits the data best.

^f^ Interaction did not include all main effects.

**TABLE A2:** Leaf‐off (November – April) and leaf‐on (May‐October) predicted probability of survival for New England cottontails (*Sylvilagus transitionalis*) and eastern cottontails (*Sylvilagus floridanus*) when covariates are held at their means

Season	Age	Body Condition	Species	Survival
Leaf‐on	Adult	Poor	Eastern cottontail	0.01 (0.00–0.15)
			New England cottontail	0.05 (0.00–0.23)
		Good	Eastern cottontail	0.64 (0.50–0.75)
			New England cottontail	0.73 (0.62–0.82)
	Juvenile	Poor	Eastern cottontail	0.49 (0.00–0.98)
			New England cottontail	0.95 (0.12–1.00)
		Good	Eastern cottontail	0.63 (0.08–0.92)
			New England cottontail	0.97 (0.65–1.00)
Leaf‐off	Adult	Poor	Eastern cottontail	0.31 (0.04–0.64)
			New England cottontail	0.44 (0.12–0.73)
		Good	Eastern cottontail	0.30 (0.20–0.41)
			New England cottontail	0.43 (0.31–0.54)

Note

95% lower and upper prediction intervals shown in brackets.
