# Supplementary material for: Is conservation based on best available science creating an ecological trap for an imperiled lagomorph?
Source: Ecol Evol. 2020 Dec 19;11(2):912–30. doi: 10.1002/ece3.7104 (PMC7820145; doi:10.1002/ece3.7104)
Supplement: Supplementary file 1 — Appendix S1 and S2 [file ECE3-11-912-s001.docx]

Appendix 1. Impacts of weather, health, and resource and landscape factors on juvenile and adult New England (*Sylvilagus transitionalis*) and eastern cottontail (*Sylvilagus floridanus*) survival in New York, 2013-2017. All considered models shown for weather, health, resource and landscape, and combined model sets. All models with interactions contained main effects unless otherwise noted.

| Age | Set | Model | K^a^ | Relative likelihood^b^ | AICc^c^ | ΔAIC_c_^d^ | *w*_i_^e^ | Deviance |
| --- | --- | --- | --- | --- | --- | --- | --- | --- |
| Juvenile | Weather | Null | 1 | 1.00 | 84.05 | 0.00 | 0.52 | 82.05 |
|  |  | Species | 2 | 0.38 | 86.00 | 1.95 | 0.19 | 81.98 |
|  |  | Species + precipitation | 3 | 0.19 | 87.35 | 3.29 | 0.10 | 81.31 |
|  |  | Species + minimum temperature | 3 | 0.18 | 87.44 | 3.38 | 0.10 | 81.40 |
|  |  | Species + year | 3 | 0.14 | 87.99 | 3.94 | 0.07 | 81.96 |
|  |  | Species + precipitation × minimum temperature | 5 | 0.05 | 90.19 | 6.14 | 0.02 | 80.10 |
|  |  |  |  |  |  |  |  |  |
|  | Body | Species + ticks × body condition | 5 | 1.00 | 70.92 | 0.00 | 0.98 | 60.82 |
|  |  | Species + ticks | 3 | 0.01 | 79.88 | 8.96 | 0.01 | 73.84 |
|  |  | Species + body condition | 3 | 0.01 | 80.66 | 9.75 | 0.01 | 74.62 |
|  |  | Null | 1 | 0.00 | 84.05 | 13.14 | 0.00 | 82.05 |
|  |  | Species + hematocrit | 3 | 0.00 | 86.92 | 16.01 | 0.00 | 80.89 |
|  |  |  |  |  |  |  |  |  |
|  | Landscape | Species + palatable stems | 3 | 1.00 | 82.78 | 0.00 | 0.33 | 76.74 |
|  |  | Species + movement distance + palatable stems | 4 | 0.62 | 83.72 | 0.95 | 0.20 | 75.66 |
|  |  | Null | 1 | 0.53 | 84.05 | 1.28 | 0.17 | 82.05 |
|  |  | Species | 2 | 0.20 | 86.00 | 3.23 | 0.07 | 81.98 |
|  |  | Species + movement distance + barberry stems × palatable stems | 6 | 0.10 | 87.37 | 4.60 | 0.03 | 75.25 |
|  |  | Species + movement distance | 3 | 0.09 | 87.52 | 4.74 | 0.03 | 81.48 |
|  |  | Species + patch area | 3 | 0.09 | 87.67 | 4.89 | 0.03 | 81.63 |
|  |  | Species + barberry stems | 3 | 0.08 | 87.81 | 5.03 | 0.03 | 81.77 |
|  |  | Species × competition^f^ + Species | 3 | 0.07 | 88.00 | 5.22 | 0.02 | 81.96 |
|  |  | Species + canopy | 3 | 0.07 | 88.01 | 5.24 | 0.02 | 81.98 |
|  |  | Species × patch area | 4 | 0.05 | 88.83 | 6.05 | 0.02 | 80.77 |
|  |  | Species + barberry stems + movement distance | 4 | 0.04 | 89.37 | 6.59 | 0.01 | 81.30 |
|  |  | Species + barberry stems × canopy | 5 | 0.03 | 90.14 | 7.36 | 0.01 | 80.05 |
|  |  | Species + barberry stems + movement distance + patch area | 5 | 0.02 | 91.10 | 8.32 | 0.01 | 81.00 |
|  |  | Species + canopy + movement distance + patch area | 5 | 0.01 | 91.23 | 8.45 | 0.01 | 81.14 |
|  |  | Species + barberry stems + canopy + movement distance | 5 | 0.01 | 91.35 | 8.58 | 0.01 | 81.26 |
|  |  | Barberry stems + canopy × species | 5 | 0.01 | 91.39 | 8.62 | 0.00 | 81.30 |
|  |  | Species + patch area × total stems | 5 | 0.01 | 91.55 | 8.77 | 0.00 | 81.46 |
|  |  | Total stems × canopy + species | 5 | 0.01 | 91.92 | 9.15 | 0.00 | 81.83 |
|  |  |  |  |  |  |  |  |  |
|  | Combined | Species + ticks × body condition + palatable stems | 6 | 1.00 | 68.13 | 0.00 | 0.62 | 56.00 |
|  |  | Species + ticks × body condition + movement distance + palatable stems | 7 | 0.37 | 70.10 | 1.97 | 0.23 | 55.93 |
|  |  | Species + ticks × body condition | 5 | 0.25 | 70.92 | 2.79 | 0.15 | 60.82 |
|  |  | Null | 1 | 0.00 | 84.05 | 15.93 | 0.00 | 82.05 |
|  |  | Species | 2 | 0.00 | 86.00 | 17.88 | 0.00 | 81.98 |
|  |  |  |  |  |  |  |  |  |
| Adult | Weather | Species + snow depth + leaf-off | 4 | 1.00 | 1279.72 | 0.00 | 0.47 | 1271.72 |
|  |  | Species + snow fall + snow depth + leaf-off | 5 | 0.62 | 1280.69 | 0.96 | 0.29 | 1270.68 |
|  |  | Species + year + leaf-off + snow depth | 5 | 0.43 | 1281.42 | 1.70 | 0.20 | 1271.41 |
|  |  | Species + snow fall × maximum temperature + snow depth + leaf-off | 7 | 0.09 | 1284.58 | 4.85 | 0.04 | 1270.56 |
|  |  | Species + leaf-off | 3 | 0.00 | 1297.01 | 17.28 | 0.00 | 1291.00 |
|  |  | Species + leaf-off + year | 4 | 0.00 | 1298.91 | 19.18 | 0.00 | 1290.90 |
|  |  | Null | 1 | 0.00 | 1310.84 | 31.12 | 0.00 | 1308.84 |
|  |  |  |  |  |  |  |  |  |
|  | Body | Species + leaf-off × body condition | 5 | 1.00 | 1286.34 | 0.00 | 0.59 | 1276.33 |
|  |  | Species + leaf-off × body condition + tick season × ticks^f^ | 6 | 0.39 | 1288.20 | 1.87 | 0.23 | 1276.19 |
|  |  | Leaf-off × body condition + species + tick season × ticks^f^ × body condition | 7 | 0.15 | 1290.19 | 3.86 | 0.09 | 1276.18 |
|  |  | Species + leaf-off + body condition | 4 | 0.07 | 1291.80 | 5.46 | 0.04 | 1283.79 |
|  |  | Species + leaf-off + tick season × ticks^f^ × body condition | 6 | 0.04 | 1292.94 | 6.60 | 0.02 | 1280.93 |
|  |  | Species + leaf-off + tick season × ticks^f^ + weight + body condition | 6 | 0.03 | 1293.24 | 6.90 | 0.02 | 1281.23 |
|  |  | Species + leaf-off + tick season × ticks^f^ + weight + body condition + hematocrit | 7 | 0.01 | 1295.17 | 8.84 | 0.01 | 1281.16 |
|  |  | Species + leaf-off + weight | 4 | 0.01 | 1296.45 | 10.12 | 0.00 | 1288.45 |
|  |  | Species + leaf-off | 3 | 0.00 | 1297.01 | 10.67 | 0.00 | 1291.00 |
|  |  | Species + leaf-off + tick season × ticks^f^ × weight | 6 | 0.00 | 1298.50 | 12.17 | 0.00 | 1286.49 |
|  |  | Species + leaf-off + tick season × ticks^f^ | 4 | 0.00 | 1298.73 | 12.39 | 0.00 | 1290.72 |
|  |  | Species + leaf-off + hematocrit | 4 | 0.00 | 1298.76 | 12.42 | 0.00 | 1290.75 |
|  |  | Species + leaf-off + tick season × ticks^f^ × hematocrit | 6 | 0.00 | 1302.56 | 16.22 | 0.00 | 1290.55 |
|  |  | Null | 1 | 0.00 | 1310.84 | 24.51 | 0.00 | 1308.84 |
|  |  |  |  |  |  |  |  |  |
|  | Landscape | Movement distance + barberry stems × palatable stems + leaf-off + canopy × species + canopy × barberry stems | 10 | 1.00 | 1280.16 | 0.00 | 0.55 | 1260.14 |
|  |  | Movement distance + barberry stems × palatable stems + leaf-off + canopy × species + canopy | 9 | 0.35 | 1282.29 | 2.13 | 0.19 | 1264.27 |
|  |  | Leaf-off × species × competition^f^ + movement distance + barberry stems × palatable stems + leaf-off + canopy × Species + canopy × barberry stems | 12 | 0.25 | 1282.92 | 2.75 | 0.14 | 1258.88 |
|  |  | Movement distance + leaf-off × species × competition^f^ + leaf-off + canopy × species + canopy × barberry stems | 10 | 0.10 | 1284.72 | 4.56 | 0.06 | 1264.70 |
|  |  | Leaf-off × species × competition^f^ + patch area + movement distance + hunt | 7 | 0.04 | 1286.40 | 6.24 | 0.02 | 1272.39 |
|  |  | Leaf-off × species × competition^f^ + patch area + movement distance + hunt + barberry stems × palatable stems + leaf-off + canopy × species + canopy × barberry stems | 14 | 0.04 | 1286.59 | 6.42 | 0.02 | 1258.54 |
|  |  | Leaf-off × species × competition^f^ + movement distance + leaf-off × canopy × species + total stems × canopy | 13 | 0.02 | 1288.35 | 8.19 | 0.01 | 1262.31 |
|  |  | Movement distance + patch area + leaf-off × species × competition^f^ + hunt + barberry stems × palatable stems + leaf-off + canopy × species + canopy | 13 | 0.02 | 1288.52 | 8.36 | 0.01 | 1262.48 |
|  |  | Movement distance + leaf-off × canopy × species × total stems | 17 | 0.01 | 1290.46 | 10.30 | 0.00 | 1256.39 |
|  |  | Leaf-off × species × competition^f^ + patch area + movement distance + hunt + leaf-off × canopy × species × total stems | 21 | 0.00 | 1296.05 | 15.88 | 0.00 | 1253.94 |
|  |  | Species + leaf-off | 3 | 0.00 | 1297.01 | 16.84 | 0.00 | 1291.00 |
|  |  | Barberry stems × palatable stems + leaf-off + species + canopy × species + canopy × barberry stems | 9 | 0.00 | 1297.44 | 17.28 | 0.00 | 1279.42 |
|  |  | Patch area + barberry stem s× palatable stems + leaf-off + species + canopy × species + canopy × barberry stems | 10 | 0.00 | 1299.18 | 19.02 | 0.00 | 1279.15 |
|  |  | Patch area + leaf-off + canopy × species + canopy × barberry stems | 8 | 0.00 | 1299.74 | 19.58 | 0.00 | 1283.73 |
|  |  | Leaf-off × species × competition^f^ + barberry stems × palatable stems + leaf-off + canopy × species + canopy × barberry stems | 11 | 0.00 | 1300.01 | 19.85 | 0.00 | 1277.98 |
|  |  | Leaf-off × species × competition^f^ + patch area + barberry stems × palatable stems + leaf-off + canopy × species + canopy × barberry stems | 12 | 0.00 | 1301.78 | 21.61 | 0.00 | 1277.74 |
|  |  | Leaf-off × canopy × species + total stems × canopy | 10 | 0.00 | 1302.26 | 22.10 | 0.00 | 1282.23 |
|  |  | Patch area + leaf-off × species × competition^f^ + barberry stems × palatable stems + leaf-off + canopy × species | 11 | 0.00 | 1302.96 | 22.80 | 0.00 | 1280.93 |
|  |  | Leaf-off × species × competition + patch area + leaf-off × canopy × species + total stems× canopy | 13 | 0.00 | 1305.45 | 25.29 | 0.00 | 1279.41 |
|  |  | Leaf-off × canopy × species × total stems | 16 | 0.00 | 1307.04 | 26.87 | 0.00 | 1274.97 |
|  |  | Patch area + leaf-off × canopy × species × total stems | 17 | 0.00 | 1307.85 | 27.69 | 0.00 | 1273.78 |
|  |  | Hunt + leaf-off × canopy × species × total stems | 17 | 0.00 | 1308.41 | 28.25 | 0.00 | 1274.34 |
|  |  | Leaf-off × species × competition + leaf-off × canopy × species × total stems | 18 | 0.00 | 1309.38 | 29.22 | 0.00 | 1273.30 |
|  |  | Null | 1 | 0.00 | 1310.84 | 30.68 | 0.00 | 1308.84 |
|  |  |  |  |  |  |  |  |  |
|  | Combined | Leaf-off × body condition + snow depth + movement distance + barberry stems × palatable stems + canopy × species + canopy × barberry stems | 13 | 0.70 | 1244.00 | 0.70 | 0.70 | 1217.95 |
|  |  | Leaf-off × body condition + snow depth + year + snow fall + movement distance + barberry stems × palatable stems + canopy × species + canopy × barberry stems | 15 | 0.31 | 1246.34 | 2.35 | 0.22 | 1216.29 |
|  |  | Leaf-off × body condition + tick season × ticks^f^ + snow depth + year + snow fall + movement distance + barberry stems × palatable stems + leaf-off + canopy × species + canopy × barberry stems | 16 | 0.11 | 1248.35 | 4.35 | 0.08 | 1216.29 |
|  |  | Leaf-off × body condition + tick season × ticks + year + snow fall + movement distance + barberry stems × palatable stems + leaf-off + canopy × species + canopy × barberry stems | 15 | 0.00 | 1258.25 | 14.26 | 0.00 | 1228.20 |
|  |  | Snow depth + movement distance + barberry stems × palatable stems + leaf-off + canopy × species + canopy × barberry stems | 11 | 0.00 | 1261.39 | 17.40 | 0.00 | 1239.36 |
|  |  | Snow depth + snow fall + movement distance + barberry stems × palatable stems + leaf-off + canopy × species + canopy × barberry stems | 12 | 0.00 | 1262.19 | 18.20 | 0.00 | 1238.16 |
|  |  | Snow depth + snow fall + tick season × ticks^f^ + movement distance + barberry stems × palatable stems + leaf-off + canopy × species + canopy × barberry stems | 13 | 0.00 | 1263.85 | 19.85 | 0.00 | 1237.81 |
|  |  | Leaf-off × body condition + tick season × ticks + species + snow depth + year + snow fall × maximum temperature | 11 | 0.00 | 1276.83 | 32.84 | 0.00 | 1254.80 |
|  |  | Movement distance + barberry stems × palatable stems + leaf-off + canopy × species + canopy × barberry stems | 10 | 0.00 | 1280.16 | 36.17 | 0.00 | 1260.14 |
|  |  | Species + snow depth + leaf-off + year + snow fall | 6 | 0.00 | 1282.42 | 38.43 | 0.00 | 1270.41 |
|  |  | Leaf-off × body condition + species + tick season × ticks^f^ | 6 | 0.00 | 1288.20 | 44.21 | 0.00 | 1276.19 |
|  |  | Species + leaf-off | 3 | 0.00 | 1297.01 | 53.01 | 0.00 | 1291.00 |
|  |  | Null | 1 | 0.00 | 1310.84 | 66.85 | 0.00 | 1308.84 |

a K = number of parameters in the model

b Relative Likelihood = exp (-0.5*ΔAIC_c_), the likelihood ratio of the given model to the top model

c AIC_c_ = AIC corrected for small sample sizes

d ΔAIC_c_ = difference in the AIC_c_ between a given model and the top model

e *w*_i_ = AIC_c_ weights, or the probability that of the models tested the given model fits the data best

^f^ Interaction did not include all main effects

Appendix 2. Leaf-off (November – April) and leaf-on (May-October) predicted probability of survival for New England cottontails (*Sylvilagus transitionalis*) and eastern cottontails (*Sylvilagus floridanus*) when covariates are held at their means. 95% lower and upper prediction intervals shown in brackets.

| Season | Age | Body Condition | Species | Survival |
| --- | --- | --- | --- | --- |
| Leaf-on | Adult | Poor | Eastern cottontail | 0.01 (0.00-0.15) |
|  |  |  | New England cottontail | 0.05 (0.00-0.23) |
|  |  | Good | Eastern cottontail | 0.64 (0.50-0.75) |
|  |  |  | New England cottontail | 0.73 (0.62-0.82) |
|  |  |  |  |  |
|  | Juvenile | Poor | Eastern cottontail | 0.49 (0.00-0.98) |
|  |  |  | New England cottontail | 0.95 (0.12-1.00) |
|  |  | Good | Eastern cottontail | 0.63 (0.08-0.92) |
|  |  |  | New England cottontail | 0.97 (0.65-1.00) |
|  |  |  |  |  |
| Leaf-off | Adult | Poor | Eastern cottontail | 0.31 (0.04-0.64) |
|  |  |  | New England cottontail | 0.44 (0.12-0.73) |
|  |  | Good | Eastern cottontail | 0.30 (0.20-0.41) |
|  |  |  | New England cottontail | 0.43 (0.31-0.54) |
